# Prediction of the next major outbreak of COVID-19 in Mainland China and a vaccination strategy for it

**DOI:** 10.1098/rsos.230655

**Published:** 2023-08-30

**Authors:** Yuanyuan Wu, Weike Zhou, Sanyi Tang, Robert A. Cheke, Xia Wang

**Affiliations:** ^1^ School of Mathematics and Statistics, Shaanxi Normal University, Xi'an 710119, People's Republic of China; ^2^ School of Mathematics, Northwest University, Xi'an 710127, People's Republic of China; ^3^ Natural Resources Institute, University of Greenwich at Medway, Central Avenue, Chatham Maritime, Kent ME4 4TB, UK; ^4^ Department of Infectious Disease Epidemiology, School of Public Health, Imperial College London, St Mary's Campus, Norfolk Place, London W2 1PG, UK

**Keywords:** COVID-19, age-structured model, vaccination strategy, immunity waning

## Abstract

After the widespread prevalence of COVID-19 at the end of 2022 in Mainland China, a major concern is when will the second major outbreak occur and with what prevalence and fatality rates will it be associated with, as peoples' immunity from natural infection subsides. To address this, we established an age-structured model considering vaccine and infection-derived immunity, fitted an immunity-waning curve, and calibrated the model using the epidemic and vaccination data from Hong Kong in 2022. The model and the situation of the first major epidemic in Mainland China were then used to predict the prevalence rate, fatality rate and peak time of the second wave. In addition, the controlling effects of different vaccination strategies on the second major outbreak are discussed. Finally, a characterization indicator for the level of population immunity was provided. We conclude that if the prevalence of the first major epidemic was 80%, the prevalence rate of the second major outbreak would be about 37.64%, and the peak time would have been July 2 2023. Strengthening vaccination can effectively delay the peak of the second wave of the epidemic and reduce the prevalence.

## 1. Introduction

So far, the global pandemic caused by the COVID-19 virus has lasted for more than three years. Although infections with COVID-19 have engendered an unprecedented burden on the global health system and caused major public health problems, effective methods have not been found to curb the spread of the epidemic. Due to the lack of effective vaccines, in the early stages of the COVID-19 pandemic, most countries could only apply non-pharmaceutical interventions (NPIs) to slow the spread of the virus. Such measures included wearing masks, maintaining social distance, restricting people's movements, closing public places or schools and tracking and isolating confirmed cases [1,2]. Although these measures seem to be effective in the short term, the implementation of these NPIs has not fundamentally curbed the spread of the virus. Once control is relaxed or cancelled, a new wave of infections is inevitable [3,4]. The rapid spread and severe outcomes of COVID-19 infections instigated an unprecedented pace of vaccine research and development.

Many past epidemics, such as those attributable to measles and smallpox (both caused by viruses), cholera (caused by a vibrio), and tuberculosis (caused by bacteria), have been limited by vaccinations, confirming that improving the immunity level of the population is an effective way to curb the spread of such infective agents. This is not the case for malaria, caused by protozoa, for which an effective vaccination is awaited. In view of the history of successful vaccination campaigns, research and development on COVID-19 vaccines have become particularly important [5]. Vaccines authorized and approved by the World Health Organization have been proved to have highly protective effects on COVID-19 for short periods, especially in the prevention of severe disease and death [6]. However, many studies have provided evidence supporting the conclusion that the vaccine's protection against infection will be weakened due to the decline of immunity after vaccination, which increases the uncertainty after vaccination [7–12]. In addition, because of the rapid variation of strains, the effectiveness of existing vaccines against mutant strains will be significantly reduced [13,14]. In particular, the current (2023) vaccine will have lower protection against infection with the Delta virus which appeared in 2021 and against the Omicron virus that arose in 2022 [15]. Taking the Omicron variant as an example, if two doses of vaccine are inoculated, the protection against infection will reach a peak efficacy of about 65% within 2–4 weeks, but its effectiveness will then decline exponentially with an effectiveness after 25 weeks of only 8.8% [16,17]. At the same time, studies have shown that even after vaccination with a booster vaccine, there is a phenomenon of immune waning in the protection against infection. Within 2–4 weeks of vaccination, the peak effectiveness of the booster vaccine reaches 67.2%, and then at about 10 weeks after vaccination, its effectiveness will decrease to 45.7% [16,17]. This immune-waning effect has been confirmed in many countries [18].

Due to the significant impact of non-pharmaceutical intervention measures on the social economy and people's lives, and with the continuous mutations of the virus, leading to higher infectivity rates but decreased pathogenicity, many countries have gradually relaxed NPIs [19]. However, because of breakthrough infections caused by virus mutations, the immunity caused by vaccines and natural infections will gradually wane, once the NPIs are relaxed, there will still be new multi-wave outbreaks [20,21]. However, in order to protect people's lives and health, China has, until recently, always maintained very strict NPIs. Since the initial outbreak in Wuhan, no large-scale epidemic had occurred on the Chinese Mainland before November 2022. Since November 2022, the vaccination coverage rate there is high and severe disease and fatality rates have been low, so the Comprehensive Group of the Joint Prevention and Control Mechanism of the State Council issued 20 measures and 10 new measures to further optimize the prevention and control of the epidemic on 11 November and 7 December, 2022, respectively (http://www.nhc.gov.cn). A notice on the implementation of the ‘B Class B Control' overall plan for COVID-19 infections was issued on 27 December, 2022 (http://www.nhc.gov.cn). Due to strong control measures that have protected the vast majority of people from infection in the past three years, and because the protective effect of vaccination against infection has faded, a large-scale infection is inevitable after the above policy adjustments. According to the statistics of the COVID-19 nationwide infection epidemic situation in December 2022 published by the China Center for Disease Control and Prevention (CDC), both the number of positive COVID-19 cases and the positive rate from nucleic acid tests among the reporting population in each province showed a trend of first increasing and then decreasing since 9 December 2022. The positive number reached its peak (6.94 million) on 22 December and then gradually decreased, and it fell to 15 000 on 23 January, 2023. Since then, China's COVID-19 infection rate has been at a very low level because of the high population immunity level caused by large-scale infection. However, due to the immunity waning making the prospect of achieving herd immunity difficult, bringing more uncertainty to the prevention and control of the potential new round of outbreaks of COVID-19, urgent questions that need to be addressed are when and at what scale will the next series of epidemic waves arrive, and how best to adopt vaccination strategies to effectively control these infections?

To address these questions, we first developed a mathematical model describing the immunity waning and the transmission mechanisms of COVID-19 by incorporating the vaccination age, by which we mean the time since vaccination events. As Hong Kong also adjusted its prevention and control policies in 2022 and experienced multiple waves of infection, we used the epidemic data of Hong Kong in 2022 for model fitting to determine some key parameters and then predict the next wave of COVID-19 in China. Next, we explored the impact of immunity waning on the transmission trends and how to design an effective vaccination programme to maximize the herd immunity to suppress and delay the next major outbreak.

## 2. Methods

### 2.1. Model

**Figure 1.**
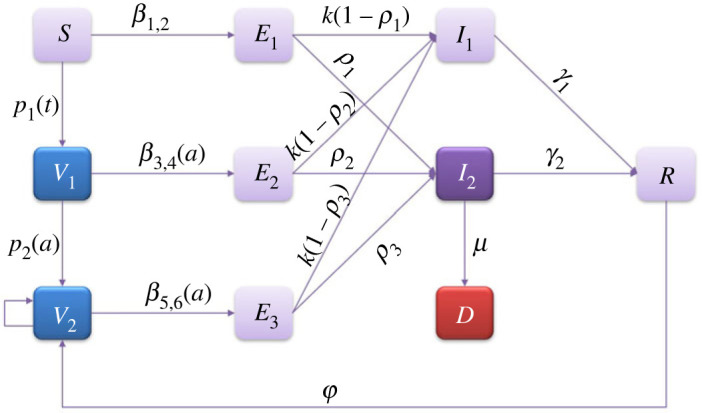
The model diagram.

In this paper, based on the transmission and vaccination process of COVID-19, and according to references [22–24], we established an age-structured infectious disease compartment model incorporating immunity waning and vaccination boosting. In the established model, susceptible individuals are divided into a non-vaccinated population (*S*), a basic immunized population (*V*_1_), and people receiving enhanced immunization (or natural infection) (*V*_2_), according to different vaccination statuses. In order to characterize the impact of different vaccination states on the severity rate, we divided the incubation compartment into three categories, the exposed population without having had any vaccination (*E*_1_), the exposed population who had received a basic vaccination (*E*_2_) and the exposed population who had received a basic vaccination and a booster vaccination (*E*_3_), respectively. According to the severity of infection, the infected individuals are divided into patients with mild symptoms (*I*_1_) and patients with severe symptoms (*I*_2_). *R* represents the population that will not be infected in the short term after recovery. The model diagram is shown in figure 1 and the equations are as follows:

2.1 $$\left\{ \begin{array}{rl} & \frac{dS}{dt}=-p_{1}(t)S(t)-\frac{\beta_{1}S(t)I_{1}(t)}{N}-\frac{\beta_{2}S(t)I_{2}(t)}{N}\;\\ & \frac{\partial V_{1}(t,a)}{\partial t}+\frac{\partial V_{1}(t,a)}{\partial a}=-p_{2}(a)V_{1}(t,a)-\frac{\beta_{3}(a)V_{1}(t,a)I_{1}(t)}{N}-\frac{\beta_{4}(a)V_{1}(t,a)I_{2}(t)}{N}\;\\ & \frac{\partial V_{2}(t,a)}{\partial t}+\frac{\partial V_{2}(t,a)}{\partial a}=-\frac{\beta_{5}(a)V_{2}(t,a)I_{1}(t)}{N}-\frac{\beta_{6}(a)V_{2}(t,a)I_{2}(t)}{N}\;\\ & \frac{dE_{1}(t)}{dt}=\frac{\beta_{1}S(t)I_{1}(t)}{N}+\frac{\beta_{2}S(t)I_{2}(t)}{N}-kE_{1}(t)\;\\ & \frac{dE_{2}(t)}{dt}=\frac{I_{1}(t)\int_{0}^{\infty }\beta_{3}(a)V_{1}(t,a)da}{N}+\frac{I_{2}(t)\int_{0}^{\infty }\beta_{4}(a)V_{1}(t,a)da}{N}-kE_{2}(t)\;\\ & \frac{dE_{3}(t)}{dt}=\frac{I_{1}(t)\int_{0}^{\infty }\beta_{5}(a)V_{2}(t,a)da}{N}+\frac{I_{2}(t)\int_{0}^{\infty }\beta_{6}(a)V_{2}(t,a)da}{N}-kE_{3}(t)\;\\ & \frac{dI_{1}(t)}{dt}=(1-\rho_{1})kE_{1}(t)+(1-\rho_{2})kE_{2}(t)+(1-\rho_{3})kE_{3}(t)-\gamma_{1}I_{1}(t)\;\\ & \frac{dI_{2}(t)}{dt}=\rho_{1}kE_{1}(t)+\rho_{2}kE_{2}(t)+\rho_{3}kE_{3}(t)-\gamma_{2}I_{2}(t)-\mu I_{2}(t)\;\\ & \frac{dR(t)}{dt}=\gamma_{1}I_{1}(t)+\gamma_{2}I_{2}(t)-\varphi R(t)\;\\ & V_{1}(t,0)=p_{1}(t)S(t)\;\\ & V_{2}(t,0)=\overset{\infty }{\underset{0}{\int }}p_{2}(a)V_{1}(t,a)da+\varphi R(t)\; \end{array}\right.$$

*β*_1_ (or *β*_2_) represents the transmission rate between *I*_1_ (or *I*_2_) and *S*. *β*_3_(*a*) (or *β*_4_(*a*)) represents the transmission rate between *I*_1_ (or *I*_2_) and *V*_1_(*t*, *a*). *β*_5_(*a*) (or *β*_6_(*a*)) represents the transmission rate between *I*_1_ (or *I*_2_) and *V*_2_(*t*, *a*). According to previous studies, the vaccination effectiveness against infection will reach its peak within 2–4 weeks, and then decline exponentially with the passage of vaccination time [16,17], so we assume that the effectiveness of vaccination (*δ_i_*, *i* = 1, 2) against infection varies as the age of vaccination increases and *β*_3_(*a*) = *β*_1_(1 − *δ*_1_(*a*)), *β*_4_(*a*) = *β*_2_(1 − *δ*_1_(*a*)), *β*_5_(*a*) = *β*_1_(1 − *δ*_2_(*a*)), *β*_6_(*a*) = *β*_2_(1 − *δ*_2_(*a*)). *p*_1_(*t*) is the vaccination rate for the basic vaccination at time *t*, and *p*_2_(*a*) is the enhanced vaccination rate for individuals with basic vaccination age *a*. 1/*k* is the incubation period. *γ*_1_ and *γ*_2_ represent the recovery rates for *I*_1_ and *I*_2_. Here, we assume that an infected person will enter the recovered compartment (*R*) after recovery, and then enter *V*_2_ at a rate δ due to the loss of immunity. Parameter definitions are shown in table 1.

**Table 1.** Definitions and values of parameters and variables. Figures in square brackets are confidence intervals.

parameters	description	value	reference
*β*_1_	the transmission rate between S and *I*_1_	0.6913[0.4294–0.8162]	estimation
*β*_2_	the transmission rate between S and*I*_2_	0.9947[0.8501–1.1783]	estimation
*ρ*_1_	severity rate without vaccination	0.3271[0.2719–0.4427]	estimation
*ρ*_2_	severity rate with basic vaccination	0.0913[0.0763–0.1416]	estimation
*ρ*_3_	severity rate with enhanced vaccination	0.0572[0.0307–0.0691]	estimation
*γ*_1_	recovery rate of *I*_1_	0.3481[0.1628–0.5074]	estimation
*γ*_2_	recovery rate of*I*_2_	0.1108[0.0627–0.2413]	estimation
μ	disease induced death rate	0.0315[0.0094–0.0517]	estimation
φ	transition rate from R to S	0.0307[0.0082–0.0384]	estimation
k	transition rate of exposed to infected individuals individualsinfected class	1/3	[25]
variables	description	initial value	reference
S	the susceptible population	4.082 × 10^6^ [4 019 819 − 4 116 700]	estimation
*V*_1_	the basic immunized population	2.254 × 10^6^	data
*V*_2_	the booster immunized population	1.036 × 10^6^	data
*E*_1_	the exposed population without vaccination	1.884 × 10^4^ [18 331 − 39 720]	estimation
*E*_2_	the exposed population with basic vaccination	734[497 − 1814]	estimation
*E*_3_	the exposed population with booster vaccination	305[196 − 865]	estimation
*I*_1_	the infected people with mild symptoms	648[422–1640]	estimation
*I*_2_	the infected people with severe symptoms	163[73–362]	estimation
R	the recovered population	2.01 × 10^3^[603–3016]	estimation

It is worth noting that according to the monthly proportion of virus strains in Hong Kong and Mainland China from January to November 2022 collected from the website of ‘Our World in Data’ (as shown in electronic supplementary material, figure S3), it can be found that the main strains circulating during this period were Omicron BA.2 and BA.5, with an average proportion of more than 89% for Omicron compared to other strains. Therefore, we neglected the evolution of toxicity differences between strains of the virus.

### 2.2. Data

**Figure 2.**
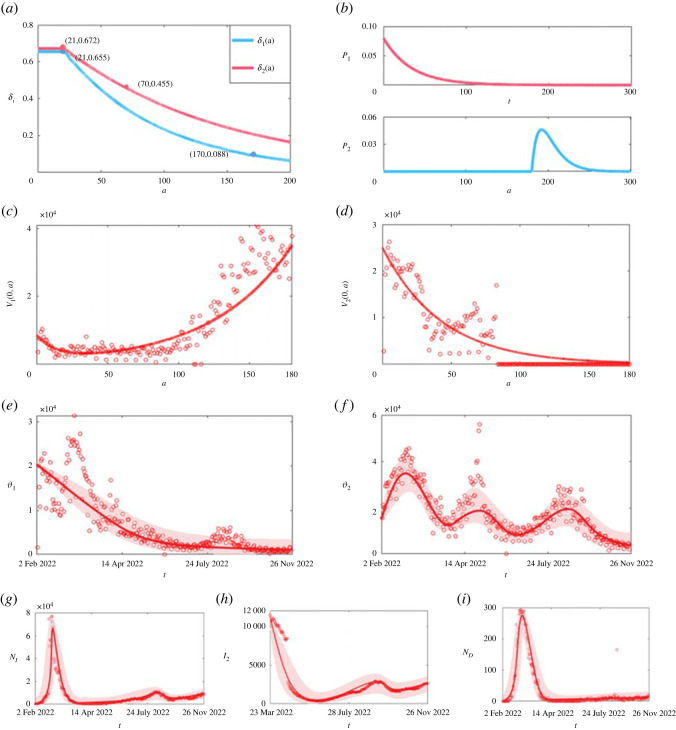
Results of model fitting. (*a*) The vaccine effectiveness versus the age of vaccination. (*b*) The vaccination rates *p*_1_(*t*) and *p*_2_(*a*). (*c,d*) The distribution of the population that has received basic vaccines and booster vaccines with vaccination age *a* at the initial time (*V*_1_(0, *a*) and *V*_2_(0, *a*), respectively. (*e*) The number of people who receive basic vaccines daily $\left(\vartheta_{1}=V_{1}(t,0)\right)$. (*f*) The number of people who receive booster vaccines daily $\left(\vartheta_{2}\right)$. (*g*) The daily number of new infections in *I*_1_. (*h*) The number of severe cases at time t (*I*_2_(*t*)). (*i*) The daily number of new deaths. The black lines are the simulation results from model (1) and the red dots are the real data.

The epidemiological data and vaccination data of COVID-19 in Hong Kong were collected from the official website of the Government of the Hong Kong Special Administrative Region (https://www.coronavirus.gov.hk/chi/5th-wave-statistics.html) and Our World In Data (https://ourworldindata.org/covid-vaccinations), from February 2022 to November 2022. The epidemiological data include the daily number of newly confirmed cases, the number of currently hospitalized cases, and the daily number of new deaths, as shown in figure 2*e*–*g*. The vaccination data include the number of people who received basic and enhanced vaccinations per day, as shown in figure 2*c*,*d*. Besides, we also collected the relevant data on the national epidemic situation of COVID-19 infection released by the Chinese CDC on 25 January 2023 (https://www.chinacdc.cn/jkzt/crb/zl/szkb_11803/), including the numbers of positive nucleic acid tests, the positive nucleic acid tests rate, the numbers of positive antigen tests and the positive antigen tests rate from 9 December 2022 to 23 January 2023, and the data on COVID-19 infected and hospitalized persons, COVID-19 positive severe patients and COVID-19 induced deaths during the period from 9 December 2022 to 23 January 2023. In addition, data on COVID-19 vaccinations in the national COVID-19 infection epidemic situation released by the Chinese CDC on 4 March 2023 were collected, including the cumulative number of monthly vaccinations against COVID-19 from December 2022 to February 2023, and the monthly coverage of the whole population's basic immunization process from December 2022 to February 2023.

### 2.3. Model calibration and parameter estimation

Multi-source data were used to calibrate the model by employing a genetic algorithm and the least-squares method. The time series data that we used included the daily number of newly confirmed cases, the number of currently hospitalized cases, the daily number of new deaths and the number of people who received basic and enhanced vaccinations per day during February 2022 to November 2022.

To calibrate the model, we firstly provide information on initial values of the variables and some parameter values by analysing the data and reviewing literature and the database, as listed in table 1. Based on the fact that there were no large-scale outbreaks in Hong Kong before 2022, and the epidemic began to rise in February 2022, we chose 2 February 2022 as the starting date of the model fitting. Considering that the booster vaccination will be administered 6 months after the basic vaccination, we push forward 6 months from the initial time, and find that the basic vaccination data increased exponentially with the vaccination time (figure 2*c*). So, we use an exponential function to fit the basic vaccination data to obtain the initial function of *V*_1_. In addition, it is found from the data that when the initial time is pushed forward for 6 months, the data on booster vaccinations are already very low. From the data, there is an exponentially decreasing relationship between booster vaccinations and the vaccination time (figure 2*d*). Therefore, an exponential function is also used to fit the booster vaccination data six months before the initial time to obtain the initial function of *V*_2_. The functions are as follows:

$$V_{1}(0,a)=w_{1}e^{z_{1}a}+w_{2}e^{z_{2}a},\;\;V_{2}(0,a)=w_{3}e^{z_{3}a},$$

where *w_i_* and *z_i_*, *i* = 1, 2, 3 are parameters to be estimated. The total population of Hong Kong is assumed to be 7 397 400 (https://www.coronavirus.gov.hk/chi/5th-wave-statistics.html) and other initial values are estimated.

Note that according to references [16,17], the effectiveness of vaccinations against infection with the Omicron variant (B.1.1.529) after receiving the basic vaccine injection reaches the maximum, approximately 65.5%, 2–4 weeks after vaccination, and then decreases with exponential trends to 8.8% by the 25th week. The protection efficiency of avoiding infection against the Omicron variant (B.1.1.529) by receiving the booster injection reaches a maximum, approximately 67.2%, 2–4 weeks after vaccination, and then decreases with exponential trends to 45.7% after 10 weeks. Thus, it is reasonable to assume that the vaccine's effectiveness peaks (*δ_i_*, *i* = 1,2) within three weeks of being given, and then decreases exponentially as the age of vaccination (i.e. the time since the vaccination) increases,

$$\delta_{i}(a)=\{\begin{array}{cc} \eta_{i}, & a<a_{i}\\ \eta_{i}e^{-r_{i}(a-a_{i})}, & a\geq a_{i} \end{array}$$

where *a* is the vaccination age, *η_i_* is the peak efficiency, *r_i_* represents the decay rate of the vaccine efficiency and *a_i_* is the time when the peak efficiency is reached and obviously *a_i_* = 21. Thus, the vaccination age-varying effectiveness *δ_i_*(*a*) of the basic vaccination and enhanced vaccination against infection could be given by estimating the values of *η_i_* and *r_i_* easily.

Furthermore, according to the data trend of the basic vaccinations, we assume that the time-varying vaccination rates *p*_1_(*t*) are describable with an exponential function as follows,

$$p_{1}(t)=m_{1}e^{n_{1}t}.$$

Besides, we assume that the vaccination rate *p*_2_(*a*) of receiving booster shots is related to the age at which the basic injection is administered. We assume that the booster injection is not administered within 6 months of receiving the basic injection and that it follows a gamma distribution after 6 months. Therefore, *p*_2_(*a*) were assumed to be

$$p_{2}(a)=\{\begin{array}{cc} 0 & a\leq 180\\ \frac{\beta^{\alpha }}{\Gamma (\alpha )}a^{\alpha -1}e^{-a\beta } & a>180 \end{array}.$$

Here, *m*_1_, *n*_1_, *α* and *β* are parameters to be estimated.

### 2.4. Initial values for prediction

According to the nationwide statistics of the COVID-19 infection epidemic situation in December (https://www.chinacdc.cn/jkzt/crb/zl/szkb_11803/), since 9 December 2022, the positive number and positive rate of COVID-19 nucleic acid tests among the reporting population in each province showed a trend of first increasing and then decreasing. The positive number reached its peak on 22 December and then gradually decreased, and it fell to 15 000 on 23 January 2023. According to the data of COVID-19 antigen detection results of the national reporting population, the number of positive antigen detections and the positive rate rose rapidly from 9 December, 2022, peaked on 22 December, then fluctuated and declined, and fell to its lowest on 23 January 2023 (https://www.chinacdc.cn/jkzt/crb/zl/szkb_11803/). Based on the above facts, the last epidemic wave in China reached its peak on December 22 2022, and basically ended on 23 January 2023 (https://www.chinacdc.cn/jkzt/crb/zl/szkb_11803/). Assuming that (a) the immunization obtained by natural infection is the same as that obtained by enhanced vaccine immunization, that (b) the total infection rate is either 70%, 80% or 90%, and (c) that the curve of daily new cases is a symmetric curve with a single peak, we use the normal distribution density function to fit the peak time and total infection rate to obtain the initial value function of *V*_2_ (see electronic supplementary material, appendix). Here, we assume that the total population in Mainland China (*N*) is 1.43 × 10^9^. So, the total population infected in the first wave was *p^I^N* , when assuming the prevalence rate to be *p^I^*.

According to the CDC's statistics on vaccination data, the coverage rate of basic vaccination by the end of November 2022 was 90.3%, and as of 2 March 2023, the coverage rate of basic vaccination was 90.6%. Assuming that the cumulative number of vaccinations per day remained the same from December 2022 to February 2023, the initial value of *V*_1_ can be obtained. Besides, according to the data reported by the China CDC, the number of COVID-19 infected persons was 248 000 on 23 January, 2023, of which 36 000 were severe cases, namely, the initial value *I*_1_(0) is 212 000, and the initial value *I*_2_(0) is 36 000. Moreover, according to the recovery rate estimated by model fitting, the initial value *R*(0) is assumed to be 74 200. Therefore, the initial number of susceptible and exposed people is (1 − *p^I^*)*N* − *I*_1_(0) − *I*_2_(0) − *R*(0). Further, according to the recovery rate and death rate of *I*_1_ and *I*_2_, we can get the average durations in the compartments of *I*_1_ and *I*_2_, respectively. So, the average number of new cases per day can be calculated to be *I*_1_(0)/*γ*_1_ and *I*_2_(0)/(*γ*_2_ + μ). Assuming that the number of new cases per day remains the same during a very short period, then

$$k[E_{1}(0)+E_{2}(0)+E_{3}(0)]=\frac{I_{1}(0)}{\gamma_{1}}+\frac{I_{2}(0)}{\left(\gamma_{2}+\mu \right)}.$$

Thus, E_1_(0) + E_2_(0) + E_3_(0) = (I_1_(0)/γ_1_ + I_2_(0)/(γ_2_ + μ))/*k*.

Then, the initial number of the susceptible compartment can be calculated from

$$S(0)=(1-p^{I})N-I_{1}(0)-I_{2}(0)-R(0)-E_{1}(0)-E_{2}(0)-E_{3}(0).$$

Due to the removal rates of *E*_1_, *E*_2_ and *E*_3_ being the same as the proportions of people in the compartments *E*_1_, *E*_2_ and *E*_3_ at time *t*, and these are the same as the proportions of people entering the three compartments per unit time,

$$\begin{array}{rl} E_{1}(0):E_{2}(0):E_{3}(0)= & \frac{\left[\beta_{1}I_{1}(0)+\beta_{2}I_{2}(0)]S(0\right)}{N}:\frac{[\beta_{1}I_{1}(0)+\beta_{2}I_{2}(0)]\int_{0}^{\infty }(1-\delta_{1}(a))V_{1}(0,a)da}{N}\\ & :\frac{[\beta_{1}I_{1}(0)+\beta_{2}I_{2}(0)]\int_{0}^{\infty }(1-\delta_{2}(a))V_{2}(0,a)da}{N}\\ & =S(0):\overset{\infty }{\underset{0}{\int }}(1-\delta_{1}(a))V_{1}(0,a)da:\overset{\infty }{\underset{0}{\int }}(1-\delta_{2}(a))V_{2}(0,a)da \end{array}$$

Thus, the initial values of the model can be given (as shown in electronic supplementary material, table S1).

## 3. Results

### 3.1. Fitting results

The vaccination age-varying effectiveness functions (*δ_i_*(*a*), *i* = 1, 2) of the basic vaccination and enhanced vaccination against infection, obtained by fitting these two variables against the age of vaccination, are shown in figure 2*a*. The effectiveness of the basic vaccination lasts about 200 days, and the effectiveness of the enhanced vaccination decays a little slower than that of the basic vaccination and lasts longer. Figure 2*b* shows the time-varying vaccination rates *p*_1_(*t*) and the age-dependent vaccination probability *p*_2_(*a*). It follows from this figure that the peak of *p*_2_(*a*) is around 190 days after the basic injection.

The fitting results of the model to the epidemic and vaccination data are given in figure 2, in which the red circles represent the true data and the black curves represent the fitted results. The estimated values of unknown parameters are listed in table 1. It follows from the following figures that the fitted curves capture the data information well. The estimation results show that the probability of developing severe symptoms without vaccination or with basic vaccination, with booster vaccination are ρ_1_ = 0.3271， ρ_2_ = 0.0913, ρ_3_ = 0.0572, respectively, revealing the effectiveness of vaccines against severe symptoms. Specifically, basic vaccination can reduce the severity rate by 72.09%, while enhanced vaccination can reduce the severity rate by 82.51%.

### 3.2. Prediction of the next major outbreak in Mainland China

**Figure 3.**
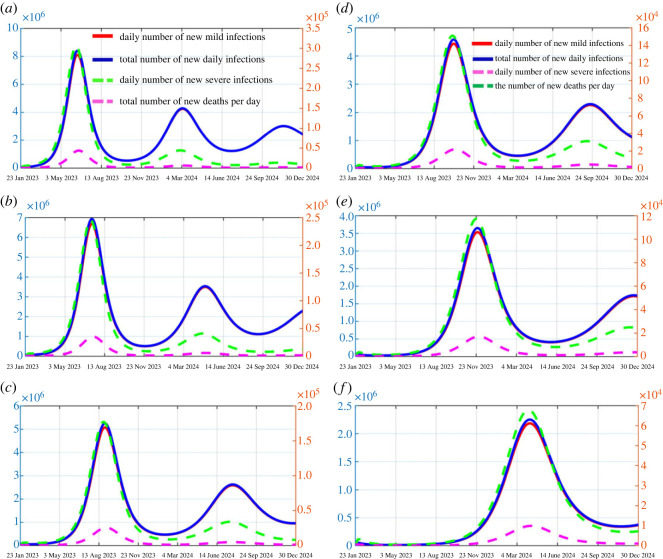
The predicted outcomes when the prevalence of the first wave is 70%, 80% and 90% are shown in (*a*), (*b*) and (*c*), respectively. The predicted results of vaccination started in mid-March, mid-April and mid-May are shown in (*d*), (*e*) and (*f*), respectively (assuming the prevalence of the first wave is 80%). The solid red curves represent the number of new mild cases per day, the solid blue curves represent the total number of new cases per day, the dotted green curves represent the number of new severe cases per day, and the dotted pink curves represent the number of new deaths per day. The solid curves correspond to the left axis, the dotted curves correspond to the right axis.

Based on the epidemic data (including epidemiological data and vaccination data) of Hong Kong, from March to December 2022, the parameters obtained from the model fitting were used to predict the epidemic trend of the COVID-19 infection in Mainland China after the implementation of ‘B class B control'. Based on the settings of the parameters and initial values shown in the methods section, we predict the COVID-19 transmission trends during the 800 days after 23 January 2023, by focusing on the daily number of new infections with mild symptoms, the daily number of new infections with severe symptoms and the daily number of new deaths. Here, we do not consider the vaccination dynamics, i.e. *p*_1_ = 0 and *p*_2_ = 0. In the following, unless otherwise stated, the starting date is 23 January 2023, and the simulation period is 800 days. Here, the prevalence of the first wave is assumed to be either 70%, 80% or 90%. The predicted results for these three scenarios are shown in table 2 and figure 3*a*–*c*.

**Table 2.** Predicted results without vaccination

prevalence rate in the first wave	70%	80%	90%
mild prevalence rate	39.81% [37.72%–43.09%]	36.36% [34.91%–42.43%]	31.59% [30.54%–37.22%]
severe prevalence rate	1.39% [1.37%–2.24%]	1.27% [1.14%–2.05%]	1.06% [1.02%–1.77%]
overall prevalence rate	41.20% [39.09%–45.33%]	37.63% [36.05%–44.48%]	32.65% [31.56%–38.99%]
fatality rate	0.21% [0.209%–0.346%]	0.19% [0.183%–0.271%]	0.16% [0.148%–0.293%]
mild peak	8.08 × 10^6^ [7 490 000–8 941 000]	6.67 × 10^6^ [4 940 000–6 841 000]	5.04 × 10^6^ [5 017 296–5 750 239]
peak severity	3.06 × 10^5^ [302 598–401 980]	2.39 × 10^5^ [225 352–351 226]	1.78 × 10^5^ [142 106–264 034]
peak total number of infections	8.39 × 10^6^ [7 792 598–9 342 980]	6.91 × 10^6^ [5 165 352–7 192 226]	5.22 × 10^6^ [5 159 402–6 014 274]
peak number of deaths	4.32 × 10^4^ [41 986–53 310]	3.51 × 10^4^ [32 964–48 890]	2.53 × 10^4^ [23 481–39 015]
peak time	09/06/2023 [17/05/2023–22/06/2023]	02/07/2023 [03/06/2023–16/07/2023]	15/08/2023 [19/07/2023–26/08/2023]

It follows from figure 3*a*–*c* that the infection peak of the next outbreak wave would occur on about 9 June, 2 July and 15 August 2023 depending on the initial prevalence, and the total infection rate of the next wave would be 41.20%, 37.63% or 32.65% under these three hypothetical scenarios (70%, 80% and 90% population had been infected during the first epidemic wave). The lower the prevalence of the first wave of the epidemic, the earlier the next wave of the epidemic arrives, and the higher the infection rate of the next wave. There are similar results for fatality rates, with the higher the prevalence in the first wave of the epidemic, the lower the fatality rate in the next wave. If the prevalence of the first wave is 70%, the fatality rate of the next wave is 0.21%, while if the prevalence of the first wave is 90%, the fatality rate of the next wave is 0.16% (reduced by 23.81%). Specifically, if the prevalence of the first wave is 80%, 37.63% of the whole population would be infected with 36.36% mild infections and 1.27% severe infections, and the fatality rate is 0.19%, in the next wave. Note that the prevalence rate in the second wave is much lower than that in the first wave (80% of the population were infected), and the mild infection is dominant in the second outbreak wave, accounting for 96.63% of the total infections. Furthermore, for the people who had been infected in the first wave, 31.46% of them would be infected in the second wave, while for the people who had not been infected in the first wave, 63.53% of them would be infected in the second wave. This implies that a large proportion of the population was effectively protected from the superinfection by obtaining immunity through natural infection in the first outbreak wave. Hence, although there will be subsequent outbreaks in the future, most of the infections are symptomatically mild and there would be fewer COVID-induced deaths. In addition, in the absence of further boosting injections, gaining immunity through natural infection could effectively reduce the superinfection risk to an extent, avoiding the large outbreak at the population level.

### 3.3. Vaccination strategy

From the above prediction results, it can be seen that the natural immunity obtained from the large-scale infections in the first wave of the epidemic has played an important role in the coming months, so that there would be no large-scale infections before May 2023. However, due to the immunity waning, a second wave of the epidemic may occur in June-August. If no extra NPIs are taken, the most direct and effective way to delay the occurrence and control the scale of the second wave of infection is to improve the level of population immunity through vaccination. Therefore, the optimized boosting programme needs to be studied further. In the following, we investigate how the mass vaccination boosting programme would affect the peak time and epidemic size of the second outbreak. In this study, we assume that vaccines are sufficient and the vaccination coverage reaches 80% in one month. To explore the critical time for activating the mass vaccination boosting programme to achieve the maximum postponement of the peak time of the second wave and minimization of the epidemic size, different activating times are chosen to investigate their impact on the peak time and the epidemic size. Due to the predicted peak time being in June to August, the activating times are chosen as mid-March, mid-April and mid-May and the simulation results are shown in figure 3*d*–*f* and electronic supplementary material, figure S1.

Figure 3*d* and table 3 show that when the prevalence of the first wave is 80%, if the boosting vaccination programme is activated in mid-March, the number of new infections reaches its maximum on 30 September 2023. The total number of infections will be 33.54% with 32.49% (accounting for 96.87%) mild infections and 1.05% severe infections (accounting for 3.13%), and the fatality rate is 0.16%. Compared with the baseline situation, the peak time is postponed for 88 days and the prevalence rate is reduced by 10.88%. Figure 3*e* shows that if the boosting vaccination programme is activated in mid-April, the number of new infections reaches a maximum on the 300th day, which corresponds to 23 November 2023. The total infection rate will be 29.42% with 28.53% mild infection rate and 0.89% severe infection rate, and the fatality rate is 0.13%. The peak infection time is further postponed over 1 month and the prevalence rate would be further reduced by 12.28%, compared with the case when boosting vaccination starts in mid-March. If the boosting vaccination programme is activated in mid-May, the infection peak of the second wave occurs on 29 March 2024. The prevalence rate is 23.79% with 23.11% mild infections and 0.68% severe infections, and the fatality rate is 0.08% (shown in figure 3*f*). The peak infection time is further postponed by four months and the prevalence rate decreases significantly.

**Table 3.** Predicted results with vaccination when the vaccination rate is 80%.

the infection rate was 80% in the vaccination situation			
vaccination time	mid-March	mid-April	mid-May
mild prevalence rate	32.49%	28.53%	23.11%
severe prevalence rate	1.05%	0.89%	0.68%
overall prevalence rate	33.54%	29.42%	23.79%
fatality rate	0.16%	0.13%	0.08%
mild peak	4.38 × 10^6^	3.51 × 10^6^	2.15 × 10^6^
peak severity	1.56 × 10^5^	1.19 × 10^5^	6.76 × 10^4^
peak total number of infections	4.54 × 10^6^	3.63 × 10^6^	2.22 × 10^6^
peak number of deaths	2.19 × 10^4^	1.70 × 10^4^	9.92 × 10^3^
peak time	30/09/2023	23/11/2023	29/03/2024

Comparing the differences in the effects of vaccination in mid-March, mid-April and mid-May on controlling the prevalence rate of the next wave of the epidemic when the prevalences of the first wave were different (table 3, electronic supplementary material, table S2 and electronic supplementary material, figure S1), we found that for all three scenarios, vaccination in mid-May was better than vaccination in mid-March and mid-April, and the prevalence and fatality rates of the next wave were the lowest. At this point, the higher the prevalence of the first wave, the lower the prevalence of the next wave. However, in the scenario where prevalence of the first wave is 70%, we find that the best vaccination implementation time is only over 20 days earlier than the peak time (9 June) of the next wave of the epidemic. In order to investigate the results of implementing vaccination later, we simulated the situation of vaccination starting on 1 June (shown in electronic supplementary material, table S2). The results showed that if the vaccination started on 1 June, the prevalence of the next wave is 38.2%, which is higher than the prevalence rate when the vaccination is implemented in mid-March. This indicates that it will be too late to implement vaccination, if the epidemic has already erupted.

In order to better describe the changes of the population immunity level over time and further explore the impact of the large-scale immunization implementation time on the subsequent development of the epidemic, we propose an indicator *L*(*t*) that characterizes the population immunity level. Here, we assume that the immunity level of *S*(*t*) is 0, the immunity level of *V_i_*(*t*, *a*) is *δ_i_*(*a*) (*i* = 1, 2), and the immunity levels of *R*(*t*), *E_i_*(*t*) and *I_i_*(*t*) (*i* = 1, 2) are 1. Then, the population immunity level at time *t* can be described as

$$L(t)=\frac{\int_{0}^{\infty }\delta_{1}(a)V_{1}(t,a)da+\int_{0}^{\infty }\delta_{2}(a)V_{2}(t,a)da+R(t)+\;E_{1}(t)+E_{2}(t)+E_{3}(t)+I_{1}(t)+I_{2}(t)}{S(t)+\int_{0}^{\infty }V_{1}(t,a)da+\int_{0}^{\infty }V_{2}(t,a)da+R(t)+\;E_{1}(t)+E_{2}(t)+E_{3}(t)+I_{1}(t)+I_{2}(t)}.$$

**Figure 4.**
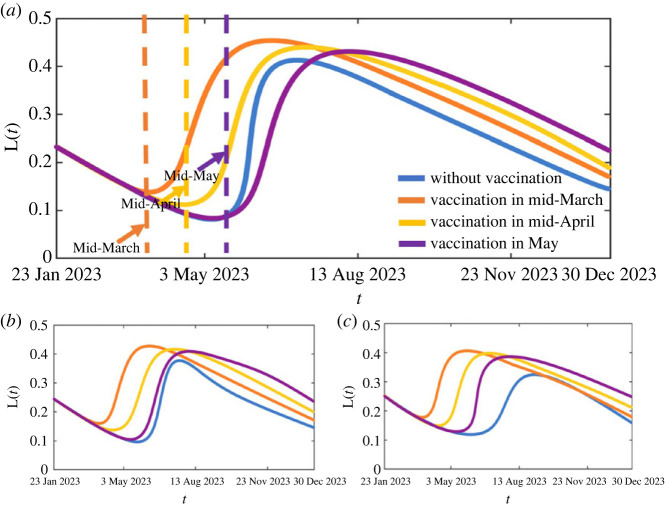
The population immunity level over time when the prevalence of the first wave is 70% (*a*), 80% (*b*) and 90% (*c*).

According to the above formula, the population immunity level without vaccination and with different vaccination times for these three scenarios are shown in figure 4. It follows from this figure that the population immunity level fluctuates over time. If vaccination is no longer carried out, the higher group immunity levels established by the previous wave of the epidemic gradually decrease over time, reaching their lowest point, and then gradually increase with the next wave of infection. Comparing three different prevalence rates (70%, 80%, 90%), the larger the prevalence of the first wave, the later the group's immune level reaches its lowest value. Specifically, if the prevalence of the first wave is 80%, *L*(*t*) will reach the lowest point on 30 May and reach the next peak on 4 July without vaccination. However, if the mass vaccination boosting programme is activated in mid-March (mid-April or mid-May), which is earlier than 30 May, *L*(*t*) will rapidly increase after the start of vaccination and reach its peak on 17 May (15 June or 14 July). As shown in figure 4, the peaks in the three cases are almost consistent, but the earlier the vaccination starts, the earlier the *L*(*t*) decreases. Similar results are observed when the prevalence of the first wave is 70% and 90%. It is worth noting that when vaccination starts in mid-May, if the first wave of infection rate is 80% or 90%, the time when vaccination starts is earlier than the time when the population's immune level reaches its lowest value. However, if the first wave of infection rate is 70% and vaccination starts in mid-May, the vaccination will start later than the time when the population's immune level reaches its minimum. So, we also calculated the situation with vaccination starting on 6 May (the lowest point of the blue line), and found that the prevalence rate was 25.87%, which is lower than the situation of vaccination starting in mid-May. The results show that with the vaccination time close to near the minimum population immunity level is most effective.

## 4. Discussion and conclusion

In order to study the impact of immunization brought by natural infection and vaccination on the transmission of COVID-19, we consider the protective effect of natural infection and vaccines on the infection and severity of the disease, and the immunity fading over time, and establish an infectious disease compartment model with vaccination age structure. The functional relationship between vaccine effectiveness and vaccination age was given, based on the effectiveness data of vaccines against infection provided in references [16,17,26]. We first used epidemic data and vaccination data from Hong Kong, China, from February to November 2022 to calibrate the model. Furthermore, based on the parameters obtained from the model calibration and combined with the initial values determined by relevant data from the China CDC, the epidemic situation in China after implementing ‘B Class B Control’ and the first large wave of infection was predicted. Finally, future vaccination strategies were discussed.

The prediction results show that, according to current control measures, the next infection peak on the Chinese Mainland is likely to occur around June-August 2023, with a prevalence rate of about 33%–41%. If the prevalence of the first wave is higher, the peak of the next wave arrives later and the prevalence and fatality rates are lower. In detail, if the prevalence of the first wave increased from 70% to 80% (or 80% to 90%), the prevalence of the next wave in Mainland China may be reduced by 8.67% (or 13.23%), and the fatality rate may be reduced by 9.52% (or 15.79%). Besides, the occurrence of the peak of the next wave may also be delayed by 25 (or 43) days. In addition, the study of the impact of large-scale vaccination on the control of the second wave indicates that activating the boosting vaccination programme could effectively delay the occurrence of the second outbreak and reduce the outbreak size. However, the effect is limited if the vaccination programme starts too early (for example, in mid-March or mid-April), since the immunity level obtained by natural infection is still high and although vaccinating would boost the immunity again, the population immunity level will decay soon. However, activating the mass booster vaccination programme in mid-May would successfully delay the next outbreak wave by about 8 months, significantly reduce the fatality rate and an extra about 10%–15% of the population could be protected from infection, whether the first wave of infection rate is 70%, 80% or 90%.

It is worth noting that we also provided characterization indicators for the level of population immunity in this study. From the perspective of population immunity level, after the first wave of infection in Mainland China, the population immunity level has dropped to about 0.23 on 23 January 2023, and continues to decline, reaching the lowest value in May. As the immune level of the population decreases, natural infections gradually increase, and the immune level caused by natural infections also increases, reaching its peak in July. The results indicate that if the population immunity level can be strengthened through vaccination near the lowest point and the total vaccination ratio reach a relatively very high level (80%), it can effectively reduce the prevalence of the next wave. However, strengthening vaccination too early or too late cannot achieve good control effects on the control of the prevalence rate in the next wave. If strengthening vaccination occurs too late, the epidemic has already erupted. If strengthening vaccination takes place too early, namely when the population's immune level is still relatively high and reinforces it again, even if the vaccination ratio reaches 50% (shown in electronic supplementary material, table S3, in the case of the prevalence of the first wave it is 80%), it can only reduce the infection rate by about 1% or 3% (mid-March or mid-April), delaying the peak of the epidemic by about half a month or nearly two months (mid-March or mid-April). Besides, even if the vaccination time is in mid-May, if the total vaccination ratio is not high enough, the control effect is still very limited (electronic supplementary material, tables S3 and S4). Therefore, considering that large-scale vaccination requires plenty of money and resources, the timing of vaccination needs to be very accurate, and the total vaccination ratio should be very high to achieve good control effects, the implementation of large-scale vaccination plans need to be very cautious.

In this paper, we use the epidemic data and vaccination data of Hong Kong in 2022 to estimate model parameters and use the epidemic data and estimated parameters of Mainland China to predict the next epidemic wave. Compared with previous studies on age-structured models [27–32], our model considers age structure more comprehensively since immunity caused by both natural infection and vaccination, the protective effect of vaccination against infection, and its protective effect against severe illness are considered. Besides, immune waning is substituted into the model through exponential functions [16,17] and, by fitting functions to the actual data, the functional expressions of an immune-waning rate that conforms to the reality are included. Compared to most studies where it is directly taken as a constant [33–38], the setting in this paper is more realistic. In addition, other function forms involved in this paper, such as the vaccine coverage rate function and the initial value function of vaccination, are fitted with the actual data to obtain the corresponding function expression, which is the highlight of this paper that is different from other similar studies [27–32]. Finally, in the vaccination strategy part, this paper also gives indicators that can represent the level of Herd immunity. This indicator can capture the change of Herd immunity level over time after vaccination, and has important reference value for the formulation of vaccination strategies and the prediction of epidemic development trends.

The models and methods established in this paper can be used for further research on the COVID-19 virus epidemic, and the characterization of population immunity level can provide a good method to support future epidemic prediction and assessment. However, due to the lack of data, some assumptions were made during the modelling process, and there are some shortcomings and areas for improvement. For example, when considering the waning mode of immunity obtained by vaccination in the article, only two data points were used to determine the immune waning function, which may cause uncertainty. When considering the immune waning mode of natural infections, it is assumed that the waning mode is consistent with the enhanced immune waning mode. Besides, the evolution of the virus and any ensuing transmissibility and virulence variations were not considered in the prediction section. These assumptions will have an impact on the results of the model, which could be improved in future research as knowledge of the immune effects caused by vaccines and natural infections continues to increase.

## Data Availability

Data and Matlab code to reproduce the analysis are available at: https://github.com/wuyuanyuan12311/SVIER_HONGKONG. The data are provided in electronic supplementary material [39].

## References

[1] Kunwar LB. 2020 Mathematical modelling of transmission dynamics of COVID-19: A case study of Nepal. Prithvi Acad. J. **3**, 19-38. (10.3126/paj.v3i1.31283)

[2] De-Leon H, Pederiva F. 2020 Particle modeling of the spreading of coronavirus disease (COVID-19). arXiv e-prints. (10.1063/5.0020565)

[3] Flaxman S et al. 2020 Estimating the effects of non-pharmaceutical interventions on COVID-19 in Europe. Nature **584**, 257-261. (10.1038/s41586-020-2405-7) 32512579

[4] Song H, Wang R, Liu S, Jin Z, He D. 2022 Global stability and optimal control for a COVID-19 model with vaccination and isolation delays. Results Phys. **42**, 106011. (10.1016/j.rinp.2022.106011) 36185819 PMC9508703

[5] Angelov G, Kovacevic R, Stilianakis NI, Veliov VM. 2023 Optimal vaccination strategies using a distributed model applied to COVID-19. Cent. Eur. J. Operat. Res. **31**, 499-521. (10.1007/s10100-022-00819-z) PMC9461439 36105892

[6] Shinde V et al. 2021 Efficacy of NVX-CoV2373 Covid-19 vaccine against the B. 1.351 variant. N. Engl. J. Med. **384**, 1899-1909. (10.1056/NEJMoa2103055) 33951374 PMC8091623

[7] Jeyanathan M, Afkhami S, Smaill F, Miller MS, Lichty BD, Xing Z. 2020 Immunological considerations for COVID-19 vaccine strategies. Nat. Rev. Immunol. **20**, 615-632. (10.1038/s41577-020-00434-6) 32887954 PMC7472682

[8] Giannitsarou C, Kissler S, Toxvaerd F. 2021 Waning immunity and the second wave: some projections for SARS-CoV-2. Am. Econ. Rev.: Insights **3**, 321-338. (10.1257/aeri.20200343)

[9] Anggriani N, Ndii MZ, Amelia R, Suryaningrat W, Pratama MAA. 2022 A mathematical COVID-19 model considering asymptomatic and symptomatic classes with waning immunity. Alexandria Eng. J. **61**, 113-124. (10.1016/j.aej.2021.04.104)

[10] Vattiato G, Lustig A, Maclaren OJ, Plank MJ. 2022 Modelling the dynamics of infection, waning of immunity and re-infection with the Omicron variant of SARS-CoV-2 in Aotearoa New Zealand. Epidemics. **41**, 100657. (10.1016/j.epidem.2022.100657) 36427472 PMC9677563

[11] Zhou W, Tang B, Bai Y, Shao Y, Xiao Y, Tang S. 2022 The resurgence risk of COVID-19 in China in the presence of immunity waning and ADE: a mathematical modelling study. Vaccine **40**, 7141-7150. (10.1016/j.vaccine.2022.10.043) 36328883 PMC9597525

[12] Gavish N, Yaari R, Huppert A, Katriel G. 2022 Population-level implications of the Israeli booster campaign to curtail COVID-19 resurgence. Sci. Transl. Med. **14**, eabn9836. (10.1126/scitranslmed.abn9836) 35412326 PMC9012104

[13] He D, Chen B, Zhao S, Stone L. 2023 The immune evasion ability of Delta variant is comparable to that of Beta variant in South Africa. BMC Public Health **23**, 1-4. (10.1186/s12889-023-15431-2) 36927400 PMC10020070

[14] Madhi SA et al. 2021 Efficacy of the ChAdOx1 nCoV-19 Covid-19 vaccine against the B.1.351 variant. N. Engl. J. Med. **384**, 1885-1898. (10.1056/NEJMoa2102214) 33725432 PMC7993410

[15] Andrews N et al. 2022 Covid-19 vaccine effectiveness against the Omicron (B.1.1.529) variant. N. Engl. J. Med. **386**, 1532-1546. (10.1056/NEJMoa2119451) 35249272 PMC8908811

[16] Szanyi J, Wilson T, Scott N, Blakely T. 2022 A log-odds system for waning and boosting of COVID-19 vaccine effectiveness. Vaccine **40**, 3821-3824. (10.1016/j.vaccine.2022.05.039) 35643564 PMC9119963

[17] Risk M, Hayek SS, Schiopu E, Yuan L, Shen C, Shi X, Freed G, Zhao L. 2022 COVID-19 vaccine effectiveness against omicron (B. 1.1. 529) variant infection and hospitalisation in patients taking immunosuppressive medications: a retrospective cohort study. Lancet Rheumatol. **4**, e775-e784. (10.1016/S2665-9913(22)00216-8) 35991760 PMC9381025

[18] Rabiu M, Iyaniwura SA. 2022 Assessing the potential impact of immunity waning on the dynamics of COVID-19 in South Africa: an endemic model of COVID-19. Nonlinear Dyn. **109**, 203-223. (10.1007/s11071-022-07225-9) 35095199 PMC8788409

[19] Fair KR, Karatayev VA, Anand M, Bauch CT. 2022 Estimating COVID-19 cases and deaths prevented by non-pharmaceutical interventions, and the impact of individual actions. Epidemics **39**, 100557. (10.1016/j.epidem.2022.100557) 35430552 PMC8985422

[20] Barthwal S et al. 2023 Neutralizing possibilities of whole virion and mRNA vaccine triggered antibodies of Wuhan strain of SARS-CoV-2 with receptor binding domains of spike proteins of Delta and Omicron strains. Asian Pacific J. Trop. Med. **16**, 92-94.

[21] Parchwani D, Dholariya S, Katoch CDS, Singh R. 2022 Growth differentiation factor 15 as an emerging novel biomarker in SARS-CoV-2 infection. World J. Methodol. **12**, 438-447. (10.5662/wjm.v12.i5.438) 36186744 PMC9516548

[22] Shen M, Xiao Y, Rong L. 2015 Global stability of an infection-age structured HIV-1 model linking within-host and between-host dynamics. Math. Biosci. Eng. **263**, 37-50. (10.1016/j.mbs.2015.02.003) 25686694

[23] Bin C, Hai FH, Hong X. 2017 Global stability of an age-structure epidemic model with imperfect vaccination and relapse. Stat. Mech. Appl. **486**, 638-655. (10.1016/j.physa.2017.05.056)

[24] Wu P, Ahmed S, Wang X, Wang H. 2023 PrEP Intervention in the mitigation of HIV epidemics in China via a data-validated age-structured model. Bull. Math. Biol. **85**, 41. (10.1007/s11538-023-01145-4) 37039932

[25] Abbott S, Sherratt K, Gerstung M, Funk S. 2022 Estimation of the test to test distribution as a proxy for generation interval distribution for the Omicron variant in England. medRxiv. (10.1101/2022.01.08.22268920)

[26] Sasanami M, Fujimoto M, Kayano T, Hayashi K, Nishiura H. 2023 Projecting the COVID-19 immune landscape in Japan in the presence of waning immunity and booster vaccination. J. Theor. Biol. **559**, 111384. (10.1016/j.jtbi.2022.111384) 36528092 PMC9749381

[27] Wang L, Liu Z, Zhang X. 2016 Global dynamics for an age-structured epidemic model with media impact and incomplete vaccination. Nonlinear Anal. Real World Appl. **32**, 136-158. (10.1016/j.nonrwa.2016.04.009)

[28] Lin J, Xu R, Tian X. 2019 Global dynamics of an age-structured cholera model with multiple transmissions, saturation incidence and imperfect vaccination. J. Biol. Dyn. **13**, 69-102. (10.1080/17513758.2019.1570362) 30696390

[29] Yu Y, Tan Y, Tang S. 2023 Stability analysis of the COVID-19 model with age structure under media effect. Comp. Appl. Math. **42**, 204. (10.1007/s40314-023-02330-w)

[30] Liu L, Xian L. 2017 Global stability of an age-structured SVEIR epidemic model with waning immunity, latency and relapse. Int. J. Biomathematics **10**, 1750038. (10.1142/S1793524517500383)

[31] Jun Y, Maia M, Lin W. 2015 Global threshold dynamics of an SIVS model with waning vaccine-induced immunity and nonlinear incidence. Math. Biosci. **268**, 1-8. (10.1016/j.mbs.2015.07.003) 26239584

[32] Cai LM, Modnak C, Wang J. 2017 An age-structured model for cholera control with vaccination. Appl. Math. Comput. **299**, 127-140. (10.1016/j.amc.2016.11.013)

[33] Luebben G, González PG, Cervantes B. 2023 Study of optimal vaccination strategies for early COVID-19 pandemic using an age-structured mathematical model: a case study of the USA. Math. Biosci. Eng. **20**, 10 828-10 865. (10.3934/mbe.2023481) PMC11216547 37322963

[34] Ram V, Schaposnik LP. 2021 A modified age-structured SIR model for COVID-19 type viruses. Sci. Rep. **11**, 15194. (10.1038/s41598-021-94609-3) 34312473 PMC8313685

[35] Mairanowski F, Below D. 2021 The age-stratified analytical model for the spread of the COVID-19 epidemic. Cold Spring Harbor Laboratory Press. (10.1101/2021.07.13.21260459)

[36] Crellen T et al. 2021 Dynamics of SARS-CoV-2 with waning immunity in the UK population. Phil. Trans. R. Soc. B Biol. Sci. **376**, 20200274. (10.1098/rstb.2020.0274) PMC8165597 34053264

[37] Pérez-Alós L et al. 2022 Modeling of waning immunity after SARS-CoV-2 vaccination and influencing factors. Nat. Commun. **13**, 1614. (10.1038/s41467-022-29225-4) 35347129 PMC8960902

[38] Are EB, Song Y, Stockdale JE, Tupper P, Colijn C. 2023 COVID-19 endgame: From pandemic to endemic? Vaccination, reopening and evolution in low- and high-vaccinated populations. J. Theor. Biol. **559**, 111368. (10.1101/2021.12.18.21268002) 36436733 PMC9686052

[39] Wu Y, Zhou W, Tang S, Cheke RA, Wang X. 2023 Prediction of the next major outbreak of COVID-19 in Mainland China and a vaccination strategy for it. Figshare. (10.6084/m9.figshare.c.6805245) PMC10465198 37650063

